# GENDER-SPECIFIC EFFECTS OF IL-6 AND DECLINES IN PROCESSING SPEED AND EXECUTIVE FUNCTIONING IN OLDER ADULTS

**DOI:** 10.1093/geroni/igae098.4344

**Published:** 2024-12-31

**Authors:** Elana Gloger, Patrick Smith, Suzanne Segerstrom

**Affiliations:** Pennsylvania State University, University Park, Pennsylvania, United States; University of North Carolina - Chapel Hill, Chapel Hill, North Carolina, United States; Oregon State University, Corvallis, Oregon, United States

## Abstract

Elevated peripheral inflammatory markers among older adults associate with cognitive decline, particularly for women, for whom cognitive decline is more prevalent and rapid. Few prospective studies have examined gender-specific associations between inflammation and cognitive decline or examined stable differences vs. fluctuations in inflammation. Participants (N = 235) were older adults (Mage = 75.1, range = 60-92) from a longitudinal study of neuropsychological assessments and blood draws every six months across up to 12 years. Systemic inflammation was operationalized as serum IL-6. The Trail Making Test provided assessment of psychomotor processing speed (A) and executive functioning (B and B-A). Multilevel models tested associations between cognition and inflammation and moderation by gender. Higher stable differences and fluctuations over time in IL-6 were significantly associated with slower TMT-A performance for women and men (between-person: g = -.06, p =.04; within-person: g = -.02, p =.049). Higher IL-6 was more strongly associated with slower TMT-B and B-A performance for men (TMT-B: g =.006, p =.02; TMT-B-A: g =.008, p =.01). Older participants demonstrated slower TMT-A performance at higher IL-6 (g =.026, p =.01). Women experience higher rates of Alzheimer’s disease diagnosis and faster age-related cognitive decline, suggesting gender-specific factors related to cognition yet to be uncovered. In the present study, executive function was sensitive to changes in systemic inflammation in a gender-specific way. Gender-specific differences in neurological aging remains a critical target for future intervention research aimed at addressing preclinical AD and accelerated aging.

